# PESTICIDES: Examining DDT’s Urogenital Effects

**DOI:** 10.1289/ehp.118-a18

**Published:** 2010-01

**Authors:** Naomi Lubick

**Affiliations:** **Naomi Lubick** is a freelance science writer based in Zürich, Switzerland, and Folsom, California. She has written for *Environmental Science & Technology*, *Nature*, and *Earth*

A team of researchers has documented a variety of urogenital malformations in male babies born to women living in an area of South Africa where the potentially endocrine-disrupting pesticide DDT is still used. The team, reporting in a study published online 23 October 2009 in *BJU International*, believes the malformations may be connected to the mothers’ DDT exposure.

A global halt to DDT’s use went into force under the Stockholm Convention on Persistent Organic Pollutants in 2004. But South Africa and other signatories where malaria is endemic can continue to use DDT to control the mosquitoes that spread malaria, which the World Health Organization estimates kills more than 700,000 African children every year. Proponents claim DDT remains the most effective and inexpensive prevention against mosquito-borne malaria. However, the insects can develop resistance to this and other chemical control methods, such as pyrethroid pesticides, and Stockholm signatories that still use DDT are urged to find replacements.

In South Africa, DDT has been sprayed annually since 1945 in the Vhembe District of Limpopo Province, where the researchers focused their study. The province has the country’s highest incidence of malaria, and the district the highest prevalence. The team, led by andrologist Riana Bornman of the University of South Africa, Pretoria, used government records from 1995 to 2003 that document the villages (though not the individual homes) where DDT was sprayed indoors. Province records were lacking for the years 1980 and 1994. Team members examined more than 7,000 male and female babies born between 2004 to 2006 to local women who agreed to answer questionnaires in hospital.

The researchers found that 11% of 3,310 baby boys born in their study cohort had at least one of several urogenital malformations including hypospadias (in which the urethral opening occurs on the underside rather than the tip of the penis) or cryptorchordism (in which one or both testes remain undescended). According to the team’s analyses, mothers exposed to household DDT spraying in the five to nine years before the study began had a 33% greater chance than unexposed mothers of having a son with such defects. The researchers also saw greater risk in women who were homemakers than in mothers employed outside the home.

The rate of urogenital malformations in local baby boys is incredibly high, says Jordi Sunyer, co-director of the Centre for Research in Environmental Epidemiology in Barcelona—the global average is estimated at about 2%. This finding alone is important, Sunyer says. But mothers from unsprayed villages also gave birth to boys with nearly the same rate of urogenital malformations, at about 10%, points out Frank Sullivan, an independent consultant in toxicology at London consultancy Harrington House.

That rate points to possible confounding circumstances, Sullivan suggests: for instance, the people living in the region could be genetically prone to such abnormalities, or they may be exposed to some other environmental factor that triggers such outcomes, for example pesticide residues in foods. In fact, the team attempted to control for such factors as eating patterns and alcohol consumption. Sullivan also points out that previous generations of women in some of the villages were exposed to DDT spraying for malaria control between 1945 and 1979, raising the possibility of intergenerational impacts.

Sunyer says biological samples may be the only way to confirm a connection between exposure to DDT and birth outcomes. Concentrations of DDT and its metabolites in blood or milk collected throughout pregnancy or lactation could pinpoint exactly when mothers had the highest levels in their bodies and be compared against rates of malformations or other adverse health effects in offspring. Sunyer also wants to investigate whether mixtures of DDT and other chemical compounds might contribute to defects.

The new study is a step toward an assessment of DDT’s impacts in an effort to judge the costs and benefits of spraying, according to scientists outside the work. Sullivan points out that thousands of children in such regions would die of malaria without such DDT spraying programs. Current strategies across Africa and elsewhere include not only indoor spraying but also development of new drugs and use of insecticide-treated bed nets.

A full assessment of the costs and benefits of spraying programs has yet to be conducted, says Sunyer. A study in the May 2007 issue of the *American Journal of Epidemiology* examined the effects of DDE (a metabolite of DDT) on androgens in baby boys in Mexico, showing little effect at low levels; but author Matthew Longnecker of the National Institute of Environmental Health Sciences and colleagues suspect effects on characteristics such as penile proportions at higher exposures.

Longnecker and Bornman plan to lead a follow-up study on DDT and birth defects in South Africa. Meanwhile, a review of DDT’s health risks will be presented at the February 2010 meeting of the parties to the Stockholm convention, and later in the year the International Programme on Chemical Safety is expected to issue an updated Environmental Health Criteria monograph on the human and environmental health effects of the pesticide.

## Figures and Tables

**Figure:**
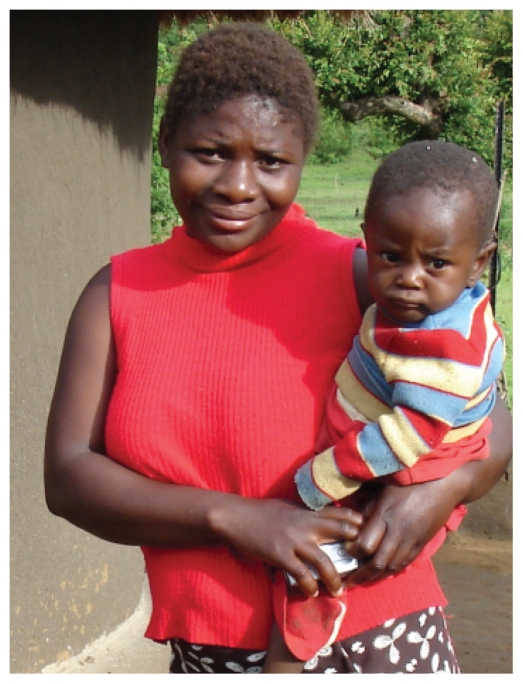
Rates of urogenital malformations among boys of Limpopo Province are more than 5 times the global average.

**Table:** Annual Use of DDT in Africa (in 10^3^ kg active ingredient)

Country	2003	2005	2007	Comment
Cameroon	0	0	0	Plan to pilot in 2009
Eritrea	13	15	15	Epidemic-prone areas
Ethiopia	272	398	371	Epidemic-prone areas
Gambia	0	0	NA	Reintroduction in 2008
Madagascar	45	0	0	Plan to resume use in 2009
Malawi	0	0	0	Plan to pilot in 2009
Mauritius	1	1	< 1	To prevent malaria introduction
Morocco	1	1	0	For occasional outbreaks
Mozambique	0	308	NA	Reintroduction in 2005
Myanmar	1	1	NA	Phasing out
Namibia	40	40	40	Long-term use
Papua New Guinea	NA	NA	0	No recent use reported
South Africa	54	62	66	Reintroduction in 2000
Sudan	75	NA	0	No recent use reported
Swaziland	NA	8	8	Long-term use
Uganda	0	0	NA	High Court prohibited use, 2008
Zambia	7	26	22	Reintroduction in 2000
Zimbabwe	0	108	12	Reintroduction in 2004

NA = not available. Adapted from van den Berg H. 2009. Global status of DDT and its alternatives for use in vector control to prevent disease. Environ Health Perspect 117:1656–1663.

